# Video-urodynamics efficacy of sacral neuromodulation for neurogenic bladder guided by three-dimensional imaging CT and C-arm fluoroscopy: a single-center prospective study

**DOI:** 10.1038/s41598-022-20731-5

**Published:** 2022-09-29

**Authors:** Shuaishuai Shan, Wen Zhu, Guoxian Zhang, Qinyong Zhang, Yingyu Che, Jianguo Wen, Qingwei Wang

**Affiliations:** Department of Urology, The First Affiliated Hospital of Zhengzhou University, No. 1, Jianshe Road, Zhengzhou, Henan China

**Keywords:** Medical research, Neurology, Urology

## Abstract

To assess the efficacy of sacral neuromodulation (SNM) for neurogenic bladder (NB), guided by intraoperative three-dimensional imaging of sacral computed tomography (CT) and mobile C-arm fluoroscopy through video-urodynamics examination. We enrolled 52 patients with NB who underwent conservative treatment with poor results between September 2019 and June 2021 and prospectively underwent SNM guided by intraoperative three-dimensional imaging of sacral CT and mobile C-arm fluoroscopy. Video-urodynamics examination, voiding diary, quality of life questionnaire, overactive bladder symptom scale (OABSS) scoring, and bowel dysfunction exam were completed and recorded at baseline, at SNM testing, and at 6-month follow-up phases. Finally, we calculated the conversion rate from period I to period II, as well as the treatment efficiency and the occurrence of adverse events during the testing and follow-up phases. The testing phase of 52 NB patients was 18–60 days, with an average of (29.3 ± 8.0) days. Overall, 38 patients underwent SNM permanent electrode implantation, whose follow-up phase was 3–25 months, with an average of (11.9 ± 6.1) months. Compared with baseline, the voiding times, daily catheterization volume, quality of life score, OABSS score, bowel dysfunction score, maximum detrusor pressure before voiding, and residual urine volume decreased significantly in the testing phase. The daily voiding volume, functional bladder capacity, maximum urine flow rate, bladder compliance, and maximum cystometric capacity increased significantly in the testing phase. Besides, the voiding times, daily catheterization volume, quality of life score, OABSS score, bowel dysfunction score, maximum detrusor pressure before voiding, and residual urine volume decreased further from the testing to follow-up phase. Daily voiding volume, functional bladder capacity, maximum urine flow rate, bladder compliance, and maximum cystometric capacity increased further from testing to follow-up. At baseline, 10 ureteral units had vesicoureteral reflux (VUR), and 9 of them improved in the testing phase. Besides, there was 1 unit that further improved to no reflux during the follow-up phase. At baseline, 10 patients had detrusor overactivity (DO), and 8 of them improved in the testing phase. Besides, 1 patient’s symptoms further improved during the follow-up phase. At baseline, there were 35 patients with detrusor-bladder neck dyssynergia (DBND); 14 (40.0%) of them disappeared during the testing phase. Among 13 cases who had DBND in the testing phase, 6 (46.2%) disappeared during the follow-up phase. Of the 47 patients with detrusor-external sphincter dyssynergia (DESD) at baseline, 8 (17.0%) disappeared during the testing phase. Among 26 cases who had DESD in the testing phase, 6 (23.1%) disappeared during the follow-up phase. The effective rate of this study was 88.5% (46/52), and the conversion rate from phase I to phase II was 73.1% (38/52). Additionally, the efficacy in a short-term follow-up was stable. SNM guided by intraoperative three-dimensional imaging of sacral CT and mobile C-arm fluoroscopy is an effective and safe treatment option for NB in short time follow-up. It would be well improved in the bladder storage function, sphincter synergetic function and emptying efficiency by video-urodynamics examination in this study.

**Trial registration**: Chinese Clinical Trial Registry. ChiCTR2100050290. Registered August 25 2021. http://www.chictr.org.cn/index.aspx.

## Introduction

Neurogenic bladder (NB) is defined as a dysfunction of the bladder and urethra caused by diseases of either the central nervous system or peripheral nerves^1^. NB is a common disease that leads to a refractory low urinary tract function and affects storage and voiding period, such as frequency, urgency, incontinence, hesitancy, weak stream, and urinary retention, which significantly affects the patient’s quality of life. NB can cause persistent intravesical hypertension and VUR, resulting in recurrent urinary tract infection, upper urinary tract dilation, and renal function impairment^2^. Moreover, conservative treatment is ineffective in some patients with NB, and augmentation enterocystoplasty can help treat patients with a small capacity and low compliance bladder effectively, especially if they have vesicoureteral reflux. However, some patients find it difficult to undergo this treatment due to the invasiveness and associated postoperative trauma^3^. Therefore, there are significant challenges in the treatment of NB.

Sacral neuromodulation (SNM) is reportedly an effective and minimally invasive method for NB or bowel dysfunction cases. However, the efficacy varies in short-term and long-term follow-up, and sacral foramen location and puncture methods are also different^4^. Furthermore, the efficacy of SNM depends on the successful passage of an electrode through the sacral foramen and optimal coupling of the target nerve, which is mainly determined by sacral foramen location and puncture method. It has been reported that the best sensory and motor nerve response is obtained at the lowest voltage for the contact point^5^.

The sacral foramen location and puncture methods for SNM include traditional methods, such as the ischial notch hand-touch location method, transcoccyx apex-measurement location method, and X-ray cross and ultrasound location method. These also include the new technology-assisted puncture methods, such as augmented reality technology and three-dimensional (3D)-printed guide-plate technology^6^. The traditional technical methods are inefficient in terms of location the sacral foramen because the patient’s position changes during operation. Moreover, some patients have sacral foramen deformities, which increase the difficulties of puncturing the sacral foramen. Additionally, the new technology-assisted puncture methods are more accurate in real-time sacral foramen positions; however, these involve significantly higher costs. However, the intraoperative three-dimensional imaging of the sacrum computed tomography (CT) combined with mobile C-arm fluoroscopy clearly showed the angle and depth of the puncture needle, helped the surgeon in deciding the needle insertion route during the operation, and guided needle insertion in real-time. CT-guidance can substantially support diagnostic and therapeutic procedures in bone and soft tissues, especially if located in deep areas of the body, which are difficult to reach using open approaches. It is reported that three-dimensional C-arm CT reformation combined with fluoroscopic guidance was a feasible, safe, and minimally invasive procedure in sacrum-related surgery.

In studies published on SNM for NB, the curative effect had been mainly assessed using a questionnaire, such as voiding diary, overactive bladder symptom scale (OABSS) score, and assessment of bowel dysfunction and quality of life. Only a few studies are based on the result of a routine urodynamic examination. The gold standard for lower urinary tract function evaluation is video-urodynamics examination according to the International Continence Society (ICS)^7^ and the International Children Continence Society^8^. Currently, only a few studies have reported the use of video-urodynamics to evaluate the effect of SNM in NB.

Therefore, this study prospectively evaluated the efficacy and safety of SNM for NB guided by intraoperative three-dimensional imaging of sacral CT and mobile C-arm fluoroscopy through video-urodynamics examination. We hope to get a good and reliable short-term effect of SNM for treating NB.

## Materials and methods

### Population

Fifty-six patients (aged 9–80 years) with NB who received SNM guided by intraoperative three-dimensional imaging of sacral CT and mobile C-arm fluoroscopy were prospectively enrolled at our hospital from September 2019 to June 2021. Four patients were lost to follow-up; one removed the electrode privately in the local hospital, and three refused to undergo video-urodynamics study examination during the testing phase. The other 52 patients showed good treatment compliance. Among the 52 patients, 19 patients had incomplete spinal cord injury; 10 patients had sacral spina bifida, 11 patients had a tethered cord, one patient had Parkinson’s disease, one patient had poliomyelitis, and 10 patients had peripheral nerve injury. Twelve of these 52 patients (23.1%) had a sacral deformity (Table 1). This study adhered to the principles of the Declaration of Helsinki, and was approved by the Ethics Committee of the First Affiliated Hospital of Zhengzhou University (Project Approval Number: 2021-KY-0138-002). All participants provided written informed consent before initiation of treatment. Inclusion criteria were as follows: (1) the presence of nervous system disease or injury, accompanied by lower urinary tract dysfunction, (2) behavioral therapy and pharmaceutical treatment were not significantly effective even after 12 weeks. (3) The patient had clinical symptoms of lower urinary tract dysfunction, such as frequency, urgency, incontinence, difficulty in voiding, or urinary retention. Exclusion criteria were organic bladder outlet obstruction, such as prostatic hyperplasia, urethral stricture, and urethral stones; complete paraplegia; and progressive neurological disease.

**Table 1:** Disease composition of 52 NB patients.

Diagnose	Cases
Incomplete spinal cord injury	19
Sacral spina bifida	10
Tethered cord	11
Parkinson’s disease	1
Poliomyelitis	1
Peripheral nerve injury	10

### Treatment procedure

Patients underwent video-urodynamics examination (model: BV Endura; Philips, Best, The Netherlands) at baseline and in SNM testing and 6-month follow-up phases to assess bladder function and the grade of VUR. The OABSS score, bowel dysfunction score, quality of life score, and voiding diary for 3 days were also recorded in all patients^9,10^.

We used the Beijing Pinchi sacral nerve electrical stimulator (Phase 1: Implantable sacral nerve stimulation electrode lead kit; Phase 2: Implantable sacral nerve stimulator kit) for SNM in this study. The specific operation steps have been reported previously^11^. The patient was lying prone, with the hips and calves raised, knee joints slightly flexed, and toes suspended. Intraoperative three-dimensional imaging of sacral CT (Model: SOMATOM Definition AS, Siemens, Erlangen, Germany) was performed to locate the precise projection points of the bilateral sacral foramen on the body surface, and an ideal puncture path was designed. After inducing local anesthesia, the puncture was guided and performed according to the designed puncture path using intraoperative three-dimensional imaging of sacral CT in real-time. This also helped confirm whether it was located in the third sacral foramen. The best sensation and motor nerve responses were obtained in the lowest voltage. Under the guidance of the mobile C-arm fluoroscopy (model: Ziehm 8000; Ziehm, Nuremberg, Germany), the depth indicator needle was placed, the introducer sleeve and the electrode of the control system were placed, and the depths were adjusted to obtain the best sensory and motor nerve response. The fixed barbed electrode was released, a subcutaneous tunnel was made, the percutaneous extension lead was inserted, and the temporary external pacemaker was finally connected.

After phase I operation, a WeChat group was established to actively follow-up with patients. Case management and nursing intervention were used to maximize the therapeutic effect of SNM. The SNM testing phase was 2–4 weeks. After this period, the effect was re-assessed using a video-urodynamics study and questionnaire, including a 3-day voiding diary, OABSS score, bowel dysfunction score, and quality of life score. NB patients often have multiple clinical symptoms at the same time, and sometimes SNM may can’t improve all clinical symptoms at one time. In view of this situation, positive changes in parameters were defined as improvement, and improvement ≥ 50% was defined as effective. If their quality of life improves by ≥ 50%, permanent electrode implantation may also be recommended. Therefore, in this study, success during testing phase was defined as all the clinical symptoms of the NB patients were effective or the quality of life was improved by ≥ 50%^12^.

The BetterStim SNM (PINS, Beijing, China) device used in the present study includes two series of implantable pulse generators (IPGs): G131 and G132; the basic components are similar to other SNM systems^13^. The stage II conversion rate refers to the percentage of patients who underwent permanent electrode implantation. The criteria for determining efficacy during the follow-up phase were the same as those during the testing phase. This study compared the differences in observation indicators above at baseline, testing, and follow-up and performed the statistical analysis. Finally, the stage II conversion rate as well as the efficiency and the occurrence of adverse events during the testing and follow-up phase, were counted. The effect was also evaluated similarly at three months follow-up after phase 2 operation.

All NB patients with VUR or residual urine volume of more than 100 mL underwent intermittent catheterization starting from SNM initiation. The frequency of catheterization depended on total daily urine output and the relative safe bladder capacity, which is the volume at the beginning of vesicoureteral reflux or cystometric detrusor pressure to 40 cmH_2_O (1 cmH_2_O = 0.098 kPa) during the video-urodynamics examination. The sum of the patient’s auto-urination volume and the residual urine volume in the empty bladder drained by intermittent catheterization must be less than the relative safe capacity every time.

The average quality of life score, OABSS score, and neurogenic bowel dysfunction score were calculated and recorded for all patients. In addition, the average daily catheterization frequency and the average daily catheterization volume were also recorded in the patients with voluntary voiding and intermittent catheterization. Additionally, the average voiding times, average voiding volume, and average functional bladder volume were recorded in patients with voluntary voiding using the voiding diary. The parameters of video-urodynamics examination included the maximum urine flow rate and residual urine volume, bladder compliance, detrusor overactivity (DO), maximum cystometric capacity, maximum detrusor pressure before voiding and maximum detrusor voiding pressure, detrusor-bladder neck dyssynergia (DBND), and detrusor-external sphincter dyssynergia (DESD). For patients with VUR, the bladder capacity and detrusor pressure at the beginning of reflux and the level of reflux throughout the examination were measured^14^.

### Statistical analysis

SPSS v21.0 statistical software (IBM Corp., Armonk, NY, USA) was used to process the data. All data were first subjected to the Shapiro‒Wilk test to determine the normality of data distribution. Normally distributed data were represented as mean ± standard deviation, and the paired samples *t*-test was used to compare the data between treatment time points. Non-normally distributed data with uneven variance are represented by mean [25th percentile, 75th percentile]. The signed-rank test was used to compare the data between treatment time points. The chi-square test was used to compare categorical data between treatment time points. Differences were considered statistically significant when P < 0.05.

### Approval of the research protocol by an institutional reviewer board

This study was performed in line with the principles of the Declaration of Helsinki, and approval was granted by the Ethics Committee of the First Affiliated Hospital of Zhengzhou University (Project Approval Number: 2021-KY-0138-002).

### Statement

All patients signed an informed consent form prior to treatment. Patients who below 16 years of age informed consent have been obtained from a parent and/or legal guardian.

### Informed consent

All eligible patients signed an informed consent form.

## Results

### Patient characteristics

The testing phase of 52 NB patients (aged 9–80 years) who underwent CT combined with C-arm guided SNM treatment ranged between 18 and 60 days, with an average of (29.3 ± 8.0) days. Their average age was (45.4 ± 18.3) years, and there were 24 females and 28 males. There were 38 patients (aged 9–80 years) who underwent SNM permanent electrode implantation; their average age was (41.6 ± 18.4) years, and 21 of these were females. Their follow-up phase ranged between 3 and 25 months, with an average of (11.9 ± 6.1) months.

### Voiding diary evaluation

The results of the voiding diary showed that the voiding times (12.1 vs. 8.3) and the daily catheterization volume (281.8 vs. 221.8 mL) decreased significantly (all P < 0.001), and the daily voiding volume (108.6 vs. 155.2 mL) and functional bladder capacity (122.3 vs. 187.9 mL) increased significantly (all P < 0.001) during testing phase compared with baseline (Table 2). Compared with the testing phase, the voiding times (8.2 vs. 7.0) and the daily catheterization volume (171.0 vs. 115.0 mL) further decreased (P < 0.001, P = 0.001) and the daily voiding volume (169.2 vs. 211.9 mL) as well as functional bladder capacity (218.5 vs. 283.3 mL) further increased (all P < 0.001) during the follow-up phase (Table 3).

**Table 2:** Comparison of data from baseline and testing phase of 52 patients with NB who underwent SNM treatment.

Parameters	Baseline	Testing phase	t/Z	*P*
Voiding times	12.1 ± 4.6	8.3 ± 2.0	4.543	< 0.001
Average voiding volume (mL)	108.6 ± 65.7	155.2 ± 95.9	− 4.259	< 0.001
Daily catheterization frequency	7.8 ± 2.8	6.7 ± 2.2	2.918	0.006
Daily catheterization volume (mL)	281.8 ± 134.9	221.8 ± 148.1	4.345	< 0.001
Functional bladder capacity (mL)	122.3 ± 78.7	187.9 ± 119.0	− 4.386	< 0.001
Quality of life score	8.7 ± 1.7	5.1 ± 2.9	8.924	< 0.001
OABSS score	4.0 [3.0, 8.0]	2.0 [1.0, 3.0]	− 4.452	< 0.001
Bowel dysfunction score	10.2 ± 5.5	5.0 ± 3.9	6.345	< 0.001
Maximum urine flow rate (mL/s)	6.7 ± 4.1	9.8 ± 6.1	− 5.849	< 0.001
Postvoid residual volumes (mL)	301.3 ± 127.1	232.4 ± 119.9	3.521	0.001
Maximum detrusor pressure before voiding (cmH_2_O)	31.7 ± 13.3	21.7 ± 11.2	3.759	0.001
Bladder compliance (mL/cmH_2_O)	9.0 ± 5.9	16.9 ± 11.8	− 3.683	0.001
Maximum cystometric capacity (mL)	227.9 ± 71.2	285.2 ± 73.6	− 4.176	< 0.001

*OABSS* overactive bladder symptom scale.

**Table 3:** Comparison of data from testing phase and follow-up phase of 38 patients with NB who underwent SNM permanent electrode implantation.

Parameters	Testing phase	Follow-up phase	t/Z	*P*
Voiding times	8.2 ± 1.8	7.0 ± 1.2	4.039	< 0.001
Average voiding volume (mL)	169.2 ± 92.3	211.9 ± 89.0	− 4.062	< 0.001
Daily catheterization frequency	6.8 ± 2.4	6.6 ± 1.6	0.738	0.469
Daily catheterization volume (mL)	171.0 [120.8, 225.0]	115.0 [36.8, 178.0]	− 3.88	0.001
Functional bladder capacity (mL)	218.5 ± 107.7	283.3 ± 98.3	− 4.884	< 0.001
Quality of life score	4.7 ± 2.7	2.6 ± 1.8	7.795	< 0.001
OABSS score	2.0 [1.0, 3.0]	1.0 [1.0, 2.0]	− 3.421	0.001
Bowel dysfunction score	5.2 ± 4.0	3.7 ± 3.1	2.843	0.015
Maximum urine flow rate (mL/s)	9.8 ± 6.1	13.5 ± 7.7	− 3.852	0.001
Postvoid residual volumes (mL)	195.0 [107.5, 312.5]	105.0 [47.5, 242.5]	− 3.414	< 0.001
Maximum detrusor pressure before voiding (cmH_2_O)	24.2 ± 10.9	16.2 ± 7.8	5.179	< 0.001
Bladder compliance (mL/cmH_2_O)	16.3 ± 11.8	25.4 ± 11.4	− 5.635	< 0.001
Maximum cystometric capacity (mL)	275.8 ± 63.4	325.1 ± 62.3	− 5.759	< 0.001

*OABSS* overactive bladder symptom scale.

### Scale score evaluation

The results of the scale score showed that the patient’s quality of life score (8.7 vs. 5.1), OABSS scores (4.0 vs. 2.0), and bowel dysfunction scores (10.2 vs. 5.0) significantly improved (all P < 0.001) during the testing phase compared with baseline (Table 2). Compared with the testing phase, the quality of life score (4.7 vs. 2.6), OABSS score (2.0 vs. 1.0), and bowel dysfunction score (5.2 vs. 3.7) improved further (P < 0.001, P = 0.001, P = 0.015) during the follow-up phase (Table 3).

### Video-urodynamics evaluation

The video-urodynamics results showed that the maximum urine flow rate (6.7 vs. 9.8 mL/s), bladder compliance (9.0 vs. 16.9 mL/cmH_2_O), and maximum cystometric capacity (227.9 vs. 285.2 mL) increased significantly, and the maximum detrusor pressure before voiding (31.7 vs. 21.7 cmH_2_O) and residual urine volume (301.3 vs. 232.4 mL) decreased significantly during testing phase compared with baseline (Table 2). Compared with the testing phase, the maximum urine flow rate (9.8 vs. 13.5 mL/s), bladder compliance (16.3 vs. 25.4 mL/cmH_2_O) and maximum cystometric capacity (275.8 vs. 325.1 mL) showed an increase and the maximum detrusor pressure before voiding (24.2 vs. 16.2 cmH_2_O) as well as the residual urine volume (195.0 vs. 105.0 mL) showed a decrease during the follow-up phase (Table 3).

**Figure 1:**
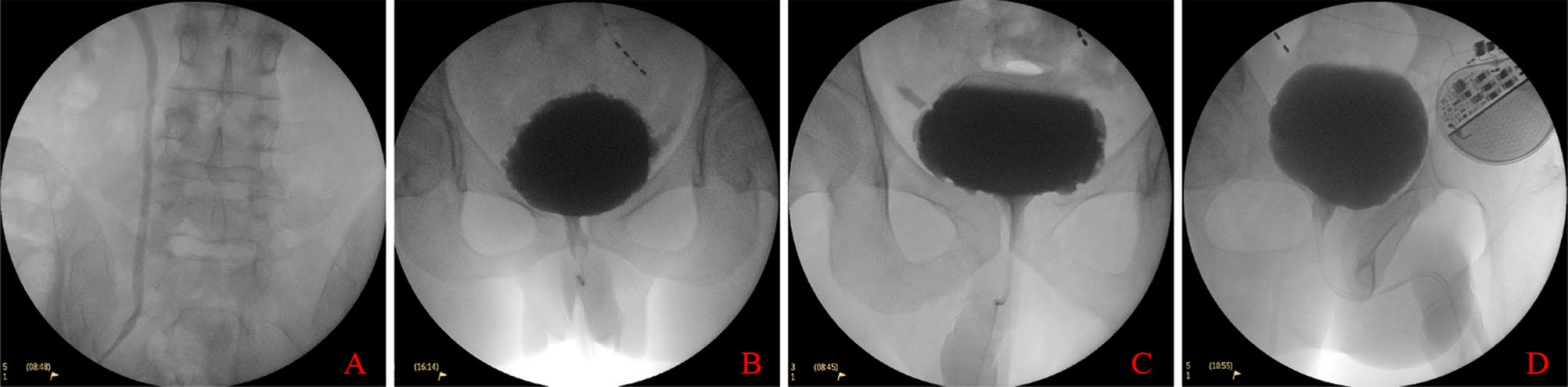
Schematic diagram of urodynamics X-ray views of 2 patients with neurogenic bladder. (**A,B**) Baseline and testing phase of the same patient, and (**A**) grade II vesicoureteral reflux (VUR) in the right ureter during storage at baseline; (**B**) no VUR in right ureter during storage in the testing phase; (**C,D**) testing phase and follow-up phase of the same patient, and (**C**) grade I VUR in right ureter during storage in the testing phase; (**B**) no VUR in right ureter during storage in the follow-up phase.

At baseline, 10 ureteral units had VUR, of which five reflux disappeared and four improved during the testing phase (Table 4). One unit further improved to no reflux during the follow-up phase (Table 5, Fig. 1). At baseline, there were 10 patients with detrusor overactivity (DO); 6 of them DO does not appear again and two of them improved during the testing phase. There was one case of DO that further improved during the follow-up phase. At baseline, there were 35 patients with detrusor-bladder neck dyssynergia (DBND); 14 (40.0%) of them improved during the testing phase. Among 13 patients who had DBND in the testing phase, 6 (46.2%) further improved during the follow-up phase. Besides, there were 47 patients with detrusor-external sphincter dyssynergia (DESD) at baseline; 8 (17.0%) of them improved during the testing phase. Among 26 patients who had DESD in the testing phase, 6 (23.1%) further improved during the follow-up phase.

**Table 4:** Improvement of VUR during the baseline and testing phase.

Patient no.	Baseline			Testing phase		
	Grade of VUR	The volume before VUR (mL)	The detrusor pressure before VUR (cmH_2_O)	Grade of VUR	The volume before VUR (mL)	The detrusor pressure before VUR (cmH_2_O)
1	Left III	50	10	–	–	–
	Right III	50	10	I	180	17
2	Right III	36	6	II	247	14
3	Right II	108	12	–	–	–
4	Left IV	190	4	IV	130	2
5	Left IV	80	8	–	–	–
6	Left III	127	37	I	257	9
7	Left II	103	29	–	–	–
8	Right I	110	26	–	–	–
9	Right II	15	5	II	209	11

**Table 5:** Improvement of VUR during the testing and follow-up phase.

Patient no.	Testing phase			Follow-up phase		
	Grade of VUR	The volume before VUR (mL)	The detrusor pressure before VUR (cmH_2_O)	Grade of VUR	The volume before VUR (mL)	The detrusor pressure before VUR (cmH_2_O)
1	Right II	209	11	II	180	15
2	Left IV	130	2	IV	170	3
3	Right I	180	17	–	–	–
4	Right II	247	14	II	145	2

In testing phase, the effective rate was 88.5% (46/52), and the conversion rate from phase I to phase II was 73.1% (38/52). The incidence of adverse events was 3.8% (2/52), which manifested as wound infection. There were four patients (10.5%) with symptom recurrence during the follow-up phase, and the symptoms improved after adjusting the stimulation parameters. Unfortunately, one patient (2.6%) had to undergo electrode removal due to capsular pouch infection. The remaining patients were stable, and there were no adverse events.

## Discussion

To date, treatment of NB remains a significant challenge owing to the effectiveness of currently available treatments, which may either exhibit moderate efficacy or be highly invasive. The FDA and China’s expert consensus does not list NB as an appropriate indication for SNM. However, many scholars have tried to use SNM to treat NB and have achieved good results^15^. SNM has the advantages of being reversible, adjustable, highly targeted, minimally invasive, and the ability to be used as staged implantation. It can improve bladder compliance and ensure that the bladder pressure is within a safe range during the storage period, thereby protecting the patient’s upper urinary tract, kidney function, partially or even completely restoring the patient’s lower urinary tract function and improving their quality of life^16^.

Engeler et al.^17^ studied 16 patients with NB caused by multiple sclerosis and showed that SNM could improve the voiding times, average voiding volume, functional bladder capacity, and postvoid residual volume. The above indicators of patients showed further improvement in the follow-up phase. The results of our study are consistent with these findings. Masood et al.^18^ analyzed 152 patients with NB from four medical centers in China who underwent SNM and found that the postvoid residual volume significantly improved during the testing phase. The postvoid residual volume in the testing phase in our study improved significantly, and the results of the two studies were consistent. Meng et al.^19^ reported that, after an average follow-up phase of 19.2 months after SNM permanent electrode implantation, the effective rate of postvoid residual volume increased from 55.6% in the testing phase to 80.0% in the follow-up phase. The improvement of postvoid residual volume showed a positive correlation with the duration of continuous regulation. The postvoid residual volume in our study was further improved during the follow-up phase, which is consistent with the results of Meng et al. Chen et al.^20^ studied 33 patients with NB caused by spina bifida and showed that SNM could improve bladder compliance, maximum cystometric capacity, and maximum detrusor pressure before urination significantly, consistent with the results of our study. Chen et al.^21^ analyzed the video-urodynamics data of 19 NB patients treated with SNM during the filling phase and found that SNM improved the bladder compliance, improved detrusor overactivity, and improved VUR in patients. In our study, there were 10 ureteral units with VUR at baseline; five of them reflux disappeared, VUR grade of two units improved from grade III to grade II and grade I, and one of them improved from grade II to grade I. One ureteral unit remained grade II VUR; however, the bladder compliance and maximum cystometric capacity increased significantly during the testing phase. During the follow-up phase, 1 ureteral unit with grade I VUR further improved to no reflux. At baseline, there were 10 patients with detrusor overactivity (DO); during the testing phase, DO disappeared in six cases. While two other patients had persistent DO, their bladder compliance and maximum cystometric capacity increased significantly during the testing phase. During the follow-up phase, VUDS examination revealed DO disappearance in 1 patient. The results of our study are consistent with the results of Chen et al.

This study showed that SNM could not only improve the urodynamic parameters of NB patients during storage but also improve urethral function during urination. At baseline, 35 patients had DBND; of these, 14 cases (40.0%) disappeared in the testing phase, and six cases (46.2%) disappeared in the follow-up phase. At baseline, there were 47 patients with DESD; of these, 8 cases (17.0%) disappeared in the testing phase, and six cases (23.1%) disappeared in the follow-up phase. Dasgupta et al.^22^ found that SNM can restore the normal perception of bladder filling in the brainstem, especially the midbrain, and control the connection between it and the cingulate gyrus of the cerebral cortex. This can regulate the urination process, thereby preventing urinary retention caused by excessive sphincter contraction. While the results of the two studies are consistent, the specific treatment mechanism described in this study needs to be further studied.

Currently, the efficacy of SNM in the treatment of NB has been confirmed by multiple centers^1^. The overall effective rate in our study was 88.5% (46/52), which is higher than the 85.0% effective rate reported by Wallace et al.^23^ and the 66.1% effective rate reported by Chaabane et al.^24^. The high effectiveness rate could be attributed to the use of intraoperative three-dimensional imaging of sacral CT and mobile C-arm fluoroscopy in our study to maximize the coupling between the electrode and the target nerve. For each patient, our team established a WeChat group to follow-up actively while applying case management nursing interventions according to the individual needs of the patient. We provided guidance to maximize the efficacy of the SNM. It is difficult for the bladder function to completely return to normal in NB patients. SNM is one of the long-term bladder function rehabilitation programs. At the end of the treatment, the bladder function of the patient is restored close to the normal bladder. The specific mechanism of successful treatment in patients with such indications remains unclear. This may be because SNM improves the normal perception and regulation of the bladder by the central nervous system, restores normal bladder function and inhibits reflex bladder activity, improves the compliance of the bladder during urine storage, reduces the protective reflex of the pelvic floor muscles, and increases contraction pressure of the bladder detrusor during urination^25^.

In this study, two patients had wound infections in the testing phase, and one of them showed purulent exudate and elevated white blood cell count. Both patients had no fever. After 7 days of anti-infective treatment combined with daily dressing change, infection symptoms improved. One patient developed wound infection in the follow-up phase, which manifested as purulent wound exudate and a fluctuating abscess, but with no capsular incision dehiscence. After 10 days of anti-infective treatment, the electrode wire was changed to the opposite side, and active measures were taken to prevent infection after the operation. However, pouch infection occurred 3 weeks after the operation, and the sacral nerve stimulator was exposed from the skin pouch. This may have been because the patient’s testing phase was extended to 67 days due to the COVID-19 pandemic. As a result, the wounds were not managed properly outside the hospital, and the patient developed scar constitution. Patients’ testing phase should be strictly controlled to 2–4 weeks^24^, and attention should be paid to the standardized management of out-of-hospital wounds and SNM infection prevention measures. In this study, the effective rate was 88.5% (46/52), and the conversion rate was 73.1% (38/52). The conversion rate was slightly lower than the effective rate because eight patients refused to undergo permanent implantation, although the treatment were effective. Among the eight patients, 4 patients refused permanent implantation due to family economic reasons, and three patients refused treatment because their expectations were too high. One other patient had the illusion that the treatment could spontaneously lead to nerve damage. Considering these factors, we stress the need for effectively communicating the treatment and its effects to the patient and family during the preoperative phase to give hope and establish reasonable expectations. At the same time, clinicians should pay attention to the patient’s family’s economic situation. The follow-up phase of this study was only 6 months, a large sample randomized controlled trial with long-term follow-up need to confirm the efficacy more accurately. Besides, SNM is not applicable to all NB patients, for example in patients with complete spinal cord injury its efficacy remains to be seen. Therefore, it is very important to select suitable NB patients for SNM.

In summary, SNM guided by intraoperative three-dimensional imaging of sacral CT and mobile C-arm fluoroscopy has a clear positive effect in the treatment of NB. Combined with intermittent hygienic catheterization, scientific and standardized management of the bladder can improve the bladder function and clinical symptoms of these patients, protect their upper urinary tract function, and improve their quality of life. The effective rate in this study was 88.5%, which could be attributed to the use of CT to guide real-time needle insertion, the establishment of an active follow-up using a WeChat group, and the implementation of a case management nursing intervention. Significant emphasis should be placed upon understanding the effectiveness of SNM. Clinicians should understand the indications for SNM and should continue to improve their theoretical knowledge and professional skills to help more NB patients.

## Data Availability

The data related to the study are available from corresponding author at request.
